# Association with Amino Acids Does Not Enhance Efficacy of Polymerized Liposomes As a System for Lung Gene Delivery

**DOI:** 10.3389/fphys.2016.00151

**Published:** 2016-04-26

**Authors:** Elga Bandeira, Miquéias Lopes-Pacheco, Nadia Chiaramoni, Débora Ferreira, Maria J. Fernandez-Ruocco, Maria J. Prieto, Tatiana Maron-Gutierrez, Ramiro M. Perrotta, Hugo C. de Castro-Faria-Neto, Patricia R. M. Rocco, Silvia del Valle Alonso, Marcelo M. Morales

**Affiliations:** ^1^Laboratory of Cellular and Molecular Physiology, Institute of Biophysics Carlos Chagas Filho, Federal University of Rio de JaneiroRio de Janeiro, Brazil; ^2^Laboratory of Pulmonary Investigation, Institute of Biophysics Carlos Chagas Filho, Federal University of Rio de JaneiroRio de Janeiro, Brazil; ^3^Laboratory of Biomembranes, Department of Science and Technology, National University of QuilmesBuenos Aires, Argentina; ^4^Laboratory of Immunopharmacology, Oswaldo Cruz Institute (FIOCRUZ)Rio de Janeiro, Brazil

**Keywords:** lung, gene delivery, mechanic parameters, nanoparticles, inflammation

## Abstract

Development of improved drug and gene delivery systems directly into the lungs is highly desirable given the important burden of respiratory diseases. We aimed to evaluate the safety and efficacy of liposomes composed of photopolymerized lipids [1,2-bis-(tricosa-10,12-diynoyl)-*sn*-glycero-3-phosphocholine] associated with amino acids as vectors for gene delivery into the lungs of healthy animals. Lipopolymer vesicles, in particular, are more stable than other types of liposomes. In this study, lipopolymers were associated with l-arginine, l-tryptophan, or l-cysteine. We hypothesized that the addition of these amino acids would enhance the efficacy of gene delivery to the lungs by the lipopolymers. l-Arginine showed the highest association efficiency due to its positive charge and better surface interactions. None of the formulations caused inflammation or altered lung mechanics, suggesting that these lipopolymers can be safely administered as aerosols. All formulations were able to induce eGFP mRNA expression in lung tissue, but the addition of amino acids reduced delivery efficacy when compared with the simple lipopolymer particle. These results indicate that this system could be further explored for gene or drug delivery targeting lung diseases.

## Introduction

Respiratory illnesses are a major health issue worldwide. Lung infections are the most important single contributor to the overall burden of disease in the world, and more than one billion people have chronic respiratory conditions (Zar and Ferkol, 2014). Despite easy access to respiratory tract, the local delivery of drugs to the lung tissue is complicated because of the unique architecture of the respiratory system and its natural defense mechanisms (Griesenbach and Alton, 2009). New therapeutic strategies have been proposed to overcome the limitations of conventional systems, including a large variety of nanoparticles.

Liposomes are small biocompatible vesicles with the ability to protect cargo from degradation, favoring controlled delivery. Their similarity to lung surfactant—both are enriched in phospholipids—makes them good candidates for lung delivery strategies (Cipolla et al., 2014). They also have the advantages of low cost and large-scale manufacturing and they are relatively easy to associate with nucleic acids.

The addition of amino acids to liposome formulations was previously tested as a method to improve drug delivery to several tissues. For example, addition of asparagine and aspartic acid improves skin permeation of several liposome formulations (Park et al., 2013). Specifically in lung tissue, association of liposomes with l-leucine was tested to deliver indomethacin, and it was found that this amino acid contributed positively to the characteristics of the powders (Chen et al., 2012). Moreover, cationic lipids, and lysine were used to enhance delivery of functional proteins into human cervical cancer cells, in the presence of serum (Sarker et al., 2014).

Our group previously tested the impact of intratracheal instillation of polymerized liposome formulations containing cationic lipids (Xisto et al., 2012). Those results showed that the formulations could induce inflammation and affected the survival of treated animals. In this work, we intended to characterize and test the safety and efficacy of polymerized liposomes carrying different amino acids (l-cysteine, l-arginine, or l-tryptophan) as a delivery system to the lungs.

## Methods

### Liposome and polymer preparation

Lipids were purchased from Avanti Polar Lipids (Alabaster, AL) and used without further purification. All solvents were of analytical grade or higher.

**Figure 1:**
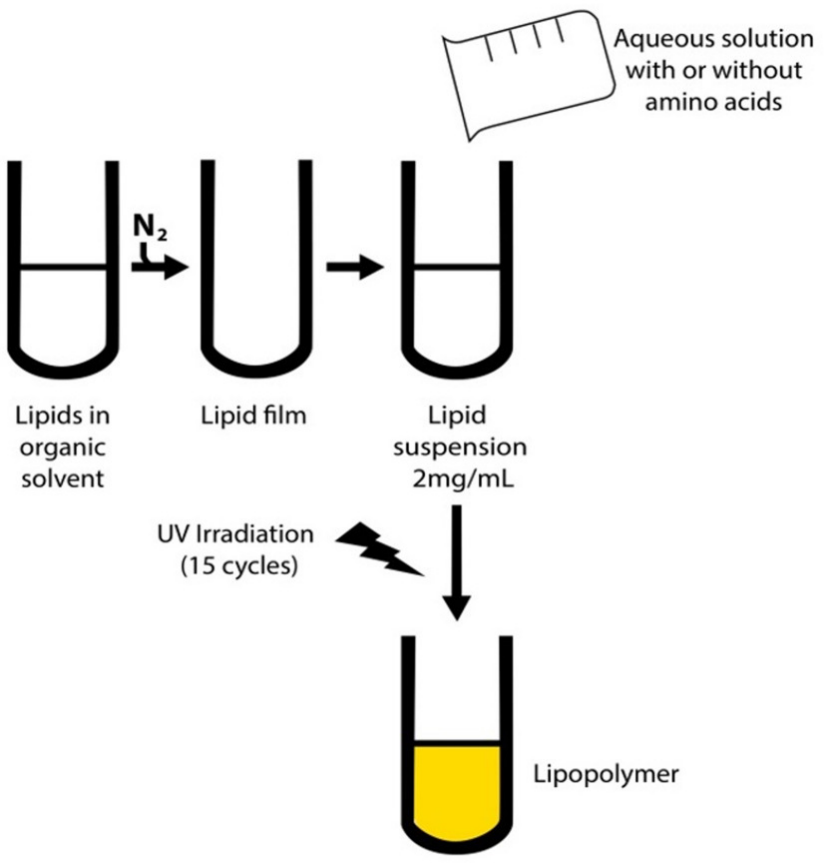
**Scheme for preparation of the lipopolymers**. First, MLVs were obtained by Bingham's method (Zar and Ferkol, 2014) and then polymerized as described previously (Griesenbach and Alton, 2009).

Lipopolymers with or without amino acids were obtained according to Figure 1. Briefly, lipids (6.4 μmol, 5.2 mg) were dissolved in chloroform; the solvent was flash-evaporated and left under vacuum for 30 min. Large multilamellar vesicles (LMVs) were obtained by adding 2 ml of phosphate-buffered saline (PBS, pH 7.4) with or without l-tryptophan, l-arginine and l-cysteine. UV irradiation was accomplished with a Stratalinker 1800 (Stratagene, La Jolla, CA) to cross-link and polymerize the diacetylenic LMVs. Polymerization was performed as previously described (Alonso-Romanowski and Chiaramoni, 2003; Chiaramoni et al., 2008, 2010; Temprana et al., 2010; Gasparri et al., 2011). Polymerization cycle energy was 360,000 mJ/cm^2^; each cycle was 1650 s in duration (15 cycles in total), at a wavelength of 254 nm. The temperature was kept at 4°C in between cycles. LMV concentration was 2 mg/ml. Visible spectra were recorded on a Shimadzu 160-A double beam spectrophotometer (Shimadzu, Kyoto, Japan) between 400 and 600 nm before and after polymerization and for non-polymerized liposomes.

### Amino acid incorporation

l-Tryptophan, l-arginine, and l-cysteine were dissolved in PBS and incorporated in the dry lipid film. The amino acid concentration was 50 mol% relative to the total lipid concentration.

### *In vitro* release of amino acids

The amino acid releasing profile of polymerized liposomes was studied in PBS as previously described (Fernandez-Ruocco et al., 2013). Non-encapsulated amino acid was separated by centrifugation. Polymerized liposomes with 50 mol% of l-tryptophan, l-arginine, or l-cysteine were prepared as described above and 0.2-ml aliquots were withdrawn at different times. The lipid suspension was incubated for 24 h at 37°C under constant agitation. After each sample was withdrawn, an aliquot of PBS was added to maintain the original concentration gradient. l-Tryptophan was quantified spectrophotometrically from the supernatant using a UV-visible spectrophotometer (Nanodrop 1000; Thermo Scientific, Wilmington, DE). Absorbance was measured at 280 nm. l-Arginine and l-cysteine were quantified colorimetrically with fluorescamine and 5,5′-dithiobis(2-nitrobenzoic acid), respectively (Miedel et al., 1989; Toyo'oka, 2009).

The release percentage was determined by the following equation:

$$\begin{array}{l} \text{TL}\left(\%\right)=\left[\left(\text{TL}t+\text{TL}t-1\right)\times 100\right]∕\text{TT} \end{array}$$

where TL*t* is the concentration of amino acid released at different times, TL*t* − 1 is the concentration of amino acid released in the previous sample (this was added to avoid error of the concentration gradient) and TT is the total amino acid concentration added to polymerized liposomes. An aliquot was withdrawn immediately after preparation and it was used to calculate the % of encapsulation. Amount of amino acid withdrawn at time *t* = 0 was taken as 100% of encapsulation. Percentages of encapsulation of the remnant aliquots were relative to the initial percentage.

### Surface modifications

To determine the spectral characteristics of the membrane/solution interface, the visible spectra of the merocyanine 540 (MC540) probe was recorded between 400 and 600 nm at 10°C to maintain the gel phase because this is the structure needed for the polymerization reaction. The MC540 probe is sensitive to the polar environment. In water, the spectra show two maxima (a dimer at 500 nm and a monomer at 530 nm). In a hydrophobic environment, the maximum is shifted toward 530 nm (MC540 dimer) and 570 nm (MC540 monomer). The first two are the characteristic absorbance maxima when the membrane is in the gel phase, whereas the second two are the characteristic absorbance maxima when the membrane is in the liquid crystalline state (Lelkes and Miller, 1980). The ratio of the absorbance at 570 nm with respect to 500 nm, called the hydrophobicity factor (HF) (Chiaramoni et al., 2008), determines the degree of hydrophobic sites exposed to the interface. The MC540 stock concentration was ~1 mg/ml. MC540, freshly prepared, was added in aliquots to give a final concentration of 10^−6^ M. The lipid/probe ratio was kept at (160:1). The sample was allowed to equilibrate for 2 min at 15°C to keep the lipids in the gel phase, and then measured. To avoid scattering, LMVs without MC540 were used as reference. The lipid concentration was kept at 2 mg/ml for the polymerization process and 0.6 mg/ml for the measurements of the visible spectra. HF was measured in samples with 15 polymerization cycles. The temperature was kept at 15°C during the measurements to ensure the gel phase.

### FTIR spectroscopy

Fourier-transform infrared (FTIR) spectroscopy was used to study the interaction between lipopolymers and amino acids as well as the changes induced by the polymerization process. To this end, 0.4 ml of the liposomal suspension with or without amino acids or polymerization was lyophilized. The solid was then resuspended in D_2_O in order to avoid interference induced by H_2_O. The lipid suspension was pipetted onto ATR cells and dried with a hair drier. Infrared spectra were recorded from 3000 to 1000 cm^−1^ in an IRAffinity-1 FTIR spectrometer (Shimadzu). Data were analyzed, normalized, and baseline corrected with the IRsolution software (Shimadzu). Frequencies were determined by the peak identification routine of the software.

### Animal preparation and experimental protocol

This study was approved by the Ethics Committee of the Institute of Biophysics Carlos Chagas Filho, Health Sciences Centre, Federal University of Rio de Janeiro. All animals received humane care in compliance with the Principles of Laboratory Animal Care formulated by the National Society for Medical Research and the Guide for the Care and Use of Laboratory Animals prepared by the National Academy of Sciences, USA.

Forty female BALB/c mice (20–25 g) were randomly assigned to five groups (*n* = 8/group). In the control group (CTRL), 50 μl of PBS was instilled intratracheally. In the lipopolymer groups, animals were instilled with lipopolymers without DNA (a 1:1 mixture of 1,2-dimyristoyl-*sn*-glycero-3-phosphocholine (DMPC)/1,2-bis-(tricosa-10,12-diynoyl)-*sn*-glycero-3-phosphocholine (DC_8, 9_PC), polymerized by 15 UV light cycles) pure (LIPO group) or conjugated with 50 mol% of l-tryptophan (TRP group), l-arginine (ARG group), or l-cysteine (CYS group) at a concentration of 2 mg/ml in 50 μl of PBS.

Animals were anesthetized with Sevoflurane (1 MAC), kept in dorsal recumbence, and then local anesthesia was performed with lidocaine (2%, 1 ml, Cristalia, Sao Paulo, SP, Brazil), a 1-cm-long midline cervical incision was made to expose the trachea, and either lipopolymers or vehicle was instilled intratracheally with an intratracheal aerosolizer (IA-1C S/M–551 Model; Penn-Century, Inc., Philadelphia, PA) attached to an FMJ-250 high-pressure syringe (Penn-Century, Inc.). The cervical incision was closed with 5.0 silk suture and the mice were returned to their cage. The animals recovered rapidly after surgery.

### Mechanical parameters

Twenty-four hours after the intra-tracheal instillation, animals were sedated (diazepam 1 mg intraperitoneally (ip), anesthetized (thiopental sodium 20 mg/kg ip), tracheotomized, paralyzed (vecuronium bromide, 0.005 mg/kg intravenously), and ventilated with a constant flow ventilator (Samay VR15; Universidad de la República, Montevideo, Uruguay) with the following parameters: frequency of 100 breaths/min, tidal volume (VT) of 0.2 ml, and fraction of inspired oxygen of 0.21. The anterior chest wall was surgically removed and a positive end-expiratory pressure of 2 cm H_2_O applied. Airflow and tracheal pressure (Ptr) were measured (Burburan et al., 2007). Lung mechanics were analyzed by the end-inflation occlusion method (Bates et al., 1988). In an open chest preparation, Ptr reflects transpulmonary pressure (PL). Briefly, after end-inspiratory occlusion, there is an initial fast drop in PL (ΔP1) from the preocclusion value down to an inflection point (Pi), followed by low pressure decay (ΔP2), until a plateau is reached.

This plateau corresponds to the elastic recoil pressure of the lung (Pel). ΔP1 selectively reflects the pressure used to overcome the airway resistance. ΔP2 reproduces the pressure spent by stress relaxation, or viscoelastic properties of the lung, together with a small contribution of pendelluft. Static lung elastance (Est) was determined by dividing Pel by VT. Lung mechanics measurements were performed 10 times per animal. All data were analyzed using ANADAT data analysis software (RHT- InfoData, Inc., Montreal, Quebec, Canada).

### Lung histology

A laparotomy was done immediately after the determination of lung mechanics and heparin (1000 IU) was injected intravenously into the vena cava. The trachea was clamped at end expiration, and the abdominal aorta and vena cava were sectioned, yielding a massive hemorrhage that quickly killed the animals. The left lung was then removed, fixed in 3% buffered formaldehyde and embedded in paraffin. Slices were cut (4-μm thick) and stained with hematoxylin–eosin. Lung histology analysis was performed with an integrating eyepiece with a coherent system consisting of a grid with 100 points and 50 lines (known length) coupled to a conventional light microscope (Olympus BX51; Olympus Latin Americas Inc., Brazil). The volume fraction of collapsed and normal pulmonary areas and the number of mononuclear and polymorphonuclear cells in pulmonary tissue was determined using the point-counting technique across 10 random, non-coincident microscopic fields (Hsia et al., 2010).

### eGFP gene delivery

Twenty female BALB/c mice were also randomly assigned to five other groups in which animals received 50 μl of *eGFP* plasmid DNA (pEGFP-C1, Vector, GenBank accession number: U55763) in PBS (CTRL) or lipopolymers conjugated with the plasmid in PBS (LIPO, CYS, TRP, or AAG) intratracheally (as described previously in animal preparation). Nine micrograms of DNA were used per dose in a final concentration of 2 mg/ml of liposomes (Xisto et al., 2012).

Twenty-four hours after delivery, the animals were euthanized and the lungs were collected, frozen in liquid nitrogen, and stored at –80°C for molecular analysis.

### qRT-PCR

Total RNA was extracted from the frozen tissues using an SV Total RNA Isolation System kit (Promega, Madison, WI) according to the manufacturer's recommendations. The concentration of RNA was measured by spectrophotometry (Nanodrop ND-1000; Thermo Scientific). First-strand cDNA was synthesized from total RNA using the GoTaq 2-Step RT-qPCR system (Promega, Madison, WI). Relative mRNA levels were measured with a SYBR green detection system using Mastercycler ep realplex (Eppendorf, Hamburg, Germany). All samples were measured in triplicate. The relative mRNA level of the *eGFP* gene was calculated as a ratio to the housekeeping gene (acidic ribosomal phosphoprotein P0; 36B4). The following primer sequences were used: eGFP: forward, 5′-CAC ATG AAG CAG CAG GAC TT-3′; reverse, 5′-GGT GCG CTC CTG GAC GTA-3′; 36B4-Rplp0: forward, 5′-CAA CCC AGC TCT GGA GAA AC-3′; reverse, 5′- GTT CTG AGC TGG CAC AGT GA-3′. Total RNA from eGFP transgenic mice was used as a positive control and taken as 100%. All results were expressed as relative to the positive control.

### Statistical analyses

Statistical analyses were run on Graphpad Prism 6.0 software (Graphpad, USA). Kolmogorov-Smirnov test was used to establish normality. One-way ANOVA followed by Dunnett's test (for comparison with lipopolymers without conjugation) or Tukey's test (for comparison among all groups) were used to compare values between groups. Values were considered significant when *p* < 0.05.

## Results

### Degree of polymerization

**Figure 2:**
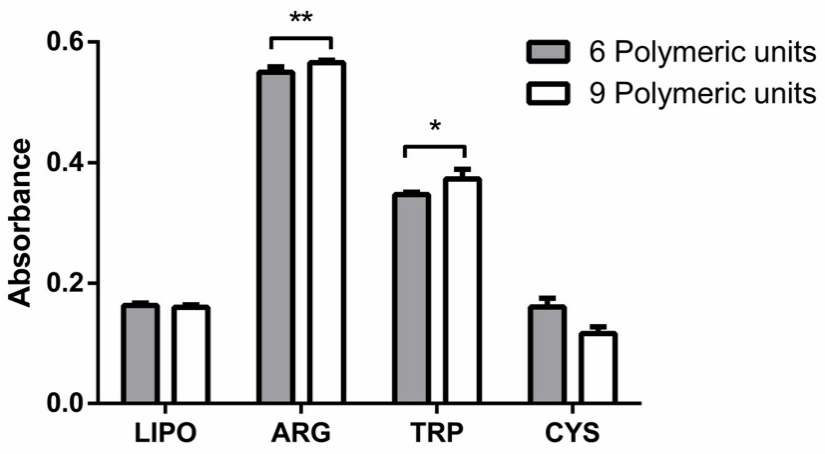
**Absorbance at 480 nm corresponding to six polymerized units conjugated effectively (gray bars) and absorbance at 520 nm corresponding to nine polymerized units conjugated effectively (white bars)**. LIPO, lipopolymer alone; ARG, TRP, CYS, lipopolymer containing l-arginine, l-tryptophan, and l-cysteine, respectively. Data are presented as mean ± SD of four independent experiments. ^*^Significant differences respect to lipopolymer without aminoacids (*P* < 0.05). ^**^Significant differences with respect to lipopolymer without aminoacids (*P* < 0.01).

To study the influence of amino acids on polymer formation, we recorded the spectra of samples with and without l-tryptophan, l-arginine, or l-cysteine. Figure 2 depicts absorbance at 480 nm (6 polymeric units) and 520 nm (9 polymeric units) of the polymerized samples.

According to the data, the amino acid that is more efficient in inducing polymerization is l-arginine. Some authors have reported that the polar heads of the phospholipids can stabilize the positive charge of l-arginine. The phosphate groups establish electrostatic interaction and hydrogen bond with the guanidinium group of the amino acid (Tsogas et al., 2005). As a result, l-arginine is inserted at the level of the polar heads, thereby increasing the compression of adjacent lipid and increasing the probability of a random encounter of the carbon chains during polymerization.

l-Tryptophan can also induce a higher concentration of polymeric units with respect to lipopolymer without amino acids. However, the effect is not as strong as that induced by l-arginine. It has been reported that l-tryptophan has a preference for the lipid interface (Esbjörner et al., 2007), but the hydrophobic character of the indole group could not induce efficient lipid packing as in the case of l-arginine. l-Tryptophan is capable of inducing closeness between carbon chains, but not as efficiently compared with l-arginine.

l-Cysteine cannot induce better polymerization efficiency in comparison with lipopolymer without amino acids. At neutral pH, l-cysteine has no charge (Veronese and Morpurgo, 1999). This could prevent its interaction with the lipid membrane, so the amino acid has no effect on polymerization efficiency.

**Figure 3:**
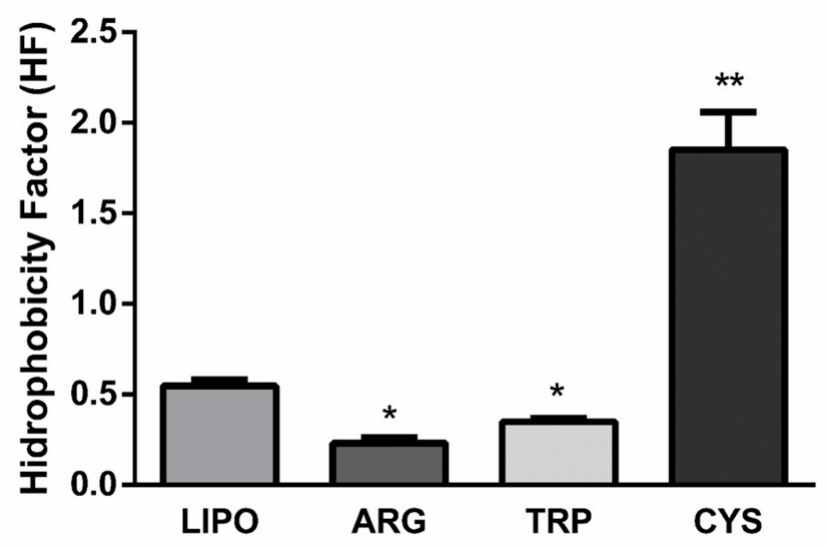
**HF (Absorbance at 570 nm/ Absorbance at 500 nm) of the lipopolymer (LIPO) or the lipopolymer with the different amino acids (ARG, TRP, CYS)**. Data are presented as mean ± *SD* of four independent experiments. ^*^Significant differences respect to lipopolymer without aminoacids (*P* < 0.05). ^**^Significant differences with respect to lipopolymer without aminoacids (*P* < 0.01).

To gain insights into the polymerization process in relation to liposome structure, particularly surface packing, the MC540 probe was used and HF was determined. As seen in Figure 3, both l-arginine and l-tryptophan can induce a decrease in HF. However, l-cysteine did not. The polymerization process itself also induces a decrease in HF. Decrease in HF is related to surface packing; a high HF means low membrane packing (Lelkes and Miller, 1980).

### *In vitro* release of amino acids

**Figure 4:**
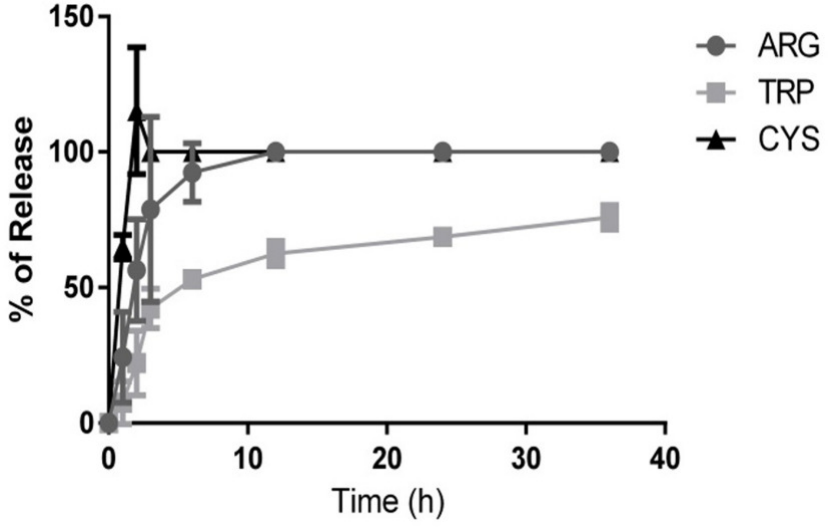
*****In vitro*** release of amino acids**. Data are presented as means ± *SD* of three independent experiments. Amino acids tested were l-cysteine (triangles), l-arginine (circles), and l-tryptophan (squares).

We determined the *in vitro* release of the three amino acids (Figure 4). l-Cysteine is released more rapidly from the bilayer than the other amino acids. It has an encapsulation efficiency of 42 ± 5%. From data corresponding to the hydrophobicity index and polymerization efficiency, we observed that this amino acid does not induce better polymerization with respect to the control and disorganizes the membrane surface. Thus, it can be suggested that, due to a less rigid surface, the amino acid cannot establish a strong interaction with the bilayer, and it is quickly released.

l-Arginine is released relatively quickly. It has an encapsulation efficiency of 45 ± 3%. The positive charge of the amino acid and the rigidity induced in the membrane after polymerization (as shown by the hydrophobicity index and polymerization efficiency results) could be responsible for the slightly more controlled release than that of l-cysteine.

l-Tryptophan has relatively low encapsulation efficiency (17 ± 1%). However, it has a controlled release for nearly 12 h.

### FTIR spectroscopy

To analyze the position of the amino acid in contact with the lipid groups, we recorded the FTIR spectra of polymerized and non-polymerized liposomes to identify different interactions that appeared to be due to the polymerization reaction.

The vibrational modes analyzed were reported by other authors (Stanish and Singh, 2001; Fournier et al., 2008). The terminal part of the head group of phosphocholine is the choline group. The vibration that corresponds to the asymmetric stretching of the ^+^N(CH_3_)_3_ is at 960–980 cm^−1^. The position of this vibration has been shown to be sensitive to dipolar interactions (Popova and Hincha, 2004). Table 1 shows the results for the FTIR experiments. The polymerization process diminishes polar interactions at the surface of the bilayer because the position of the band in the case of polymerized formulations is at higher frequencies. The addition of the three amino acids diminishes these interactions. After the polymerization process, l-arginine may induce a higher amount of dipolar interactions. This is probably because, before the polymerization process, l-arginine is mixed in the whole bilayer, but as polymerization proceeds, domains are formed and phase separation appears. l-Arginine then prefers a particular domain and increases dipolar interactions at the surface of the bilayer.

**Table 1:** **FTIR vibrational modes**.

**Vibrational modes**	**Formulations**							
	**Lipopolymer**		**Lipopolymer** + **l-arginine**		**Lipopolymer** + **l-cysteine**		**Lipopolymer** + **l-tryptophan**	
	**0 cycles**	**15 cycles**	**0 cycles**	**15 cycles**	**0 cycles**	**15 cycles**	**0 cycles**	**15 cycles**
^+^N(CH_3_)_3_	968	972	970	968	970	974	970	970
*P* = O	1244	1238	1246	1244	1246	1244	1246	1244

In the case of l-tryptophan, the amino acid is mixed in the whole bilayer and remains so after polymerization because dipolar interactions do not change after the polymerization process.

l-Cysteine interacts with the bilayer surface diminishing the dipolar interactions even more, but shown by the encapsulation efficiency, this interaction is weak.

The peak corresponding to the asymmetric stretching of the P = O bond is between 1235 and 1250 cm^−1^. The position of the peak is sensitive to the formation of H bonds or coulombic interactions shifting to lower frequencies with increasing bonding (Popova and Hincha, 2004). Table 1 presents the results of these experiments. In the case of P = O vibrational mode, the polymerization process diminishes its frequency, suggesting the presence of more interaction at the P = O level. These interactions decrease with the three amino acids before and after polymerization and suggest that the part of the amino acid that interacts with the P = O group is the same in each case.

Vibrations corresponding to the C = O group are between 1720 and 1750 cm^−1^ (Popova and Hincha, 2004). These vibrations were analyzed but there were no differences between the samples (data not shown).

### Impact of lipopolymers on lung function and inflammation

**Figure 5:**
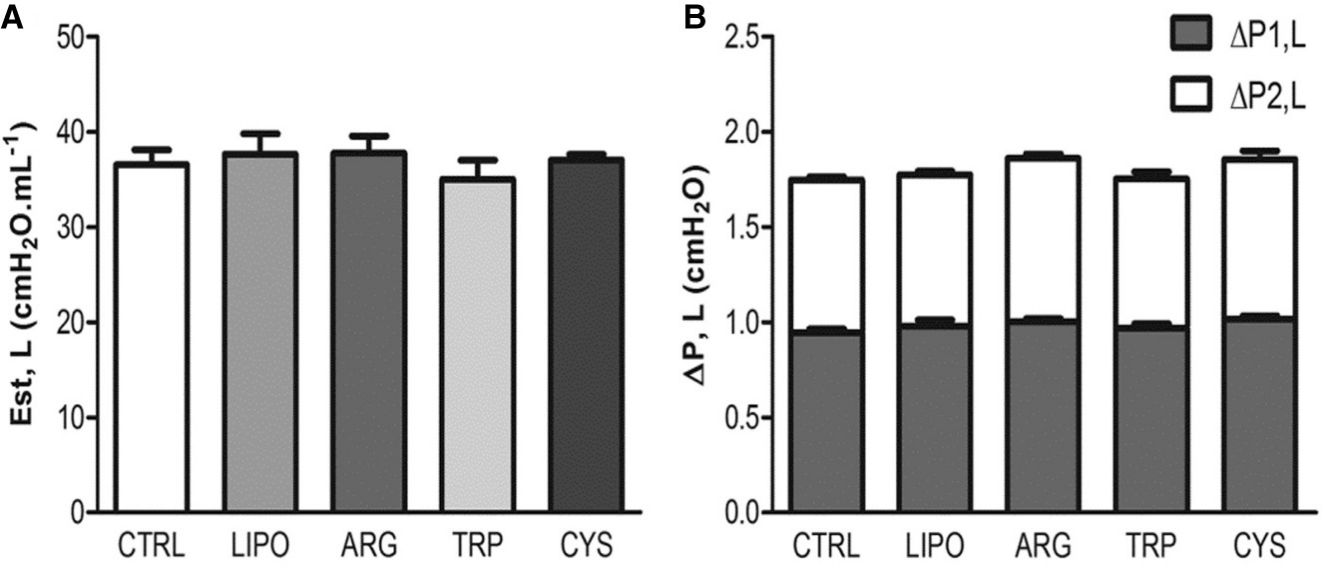
**Effects of lipopolymers on lung function**. Values are means ± *SD* of eight animals in each group (with 10 determinations per animal). Control group (CTRL) was instilled with vehicle, LIPO group was instilled with the lipopolymer only, and the ARG, TRP, and CYS groups were instilled with the lipopolymer containing l-arginine, l-tryptophan, and l-cysteine, respectively. **(A)** Lung static elastance (Est,L). **(B)** Stacked bar chart: the data in each gray bar represent the lung viscous pressure (ΔP1) and the white bars are viscoelastic/inhomogeneous (ΔP2) pressure dissipation. The whole column represents the total pressure (ΔPtot) variation in each group.

The survival rate at 24 h was 100% for all groups. Analysis of lung resistive and viscoelastic pressures and static elastance at this point showed no differences between the groups instilled with all four types of lipopolymers and the control group (Figure 5).

**Figure 6:**
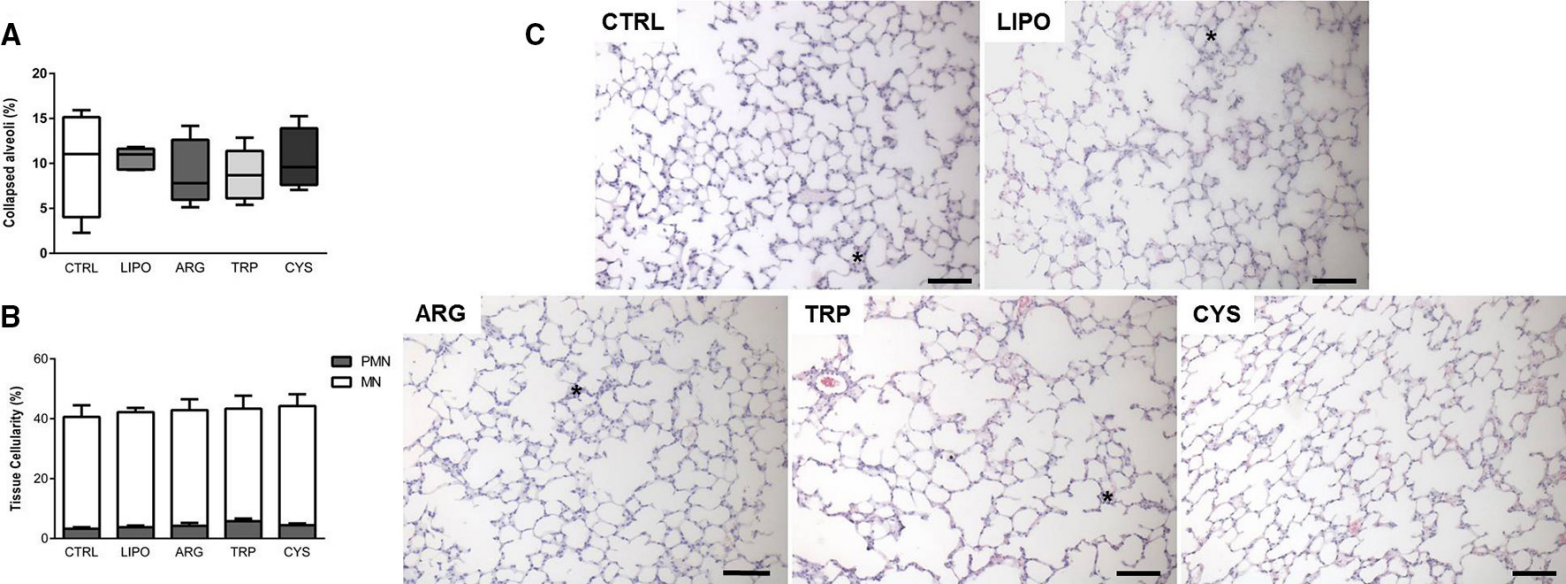
**Effects of lipopolymers on lung inflammation**. The control group (CTRL) was instilled with vehicle, the LIPO group was instilled with the lipopolymer only, and the ARG, TRP, and CYS groups were instilled with lipopolymer containing l-arginine, l-tryptophan, and l-cysteine, respectively. **(A)** Lung morphometry box plot (min. to max.). **(B)** Lung cellularity (PMN, polymorphonuclear cells; MN, mononuclear cells). Values are means ± *SD* of five animals in each group (data gathered from 10 random fields per animal). **(C)** Representative photomicrographs of lung parenchyma (×200, scale bar represents 100 μm). Asterisks indicate collapsed alveoli.

The histologic properties of the lung were assessed to evaluate lung inflammation (Figure 6C). First, we searched for alterations in lung morphology, measuring the fractional area of alveolar collapse (Figure 6A), and counting the number of inflammatory cells infiltrated in lung tissue (Figure 6B). No significant differences were observed among the groups for both parameters, suggesting a lack of inflammatory response triggered by lipopolymer instillation.

### Efficacy of *eGFP* gene delivery

**Figure 7:**
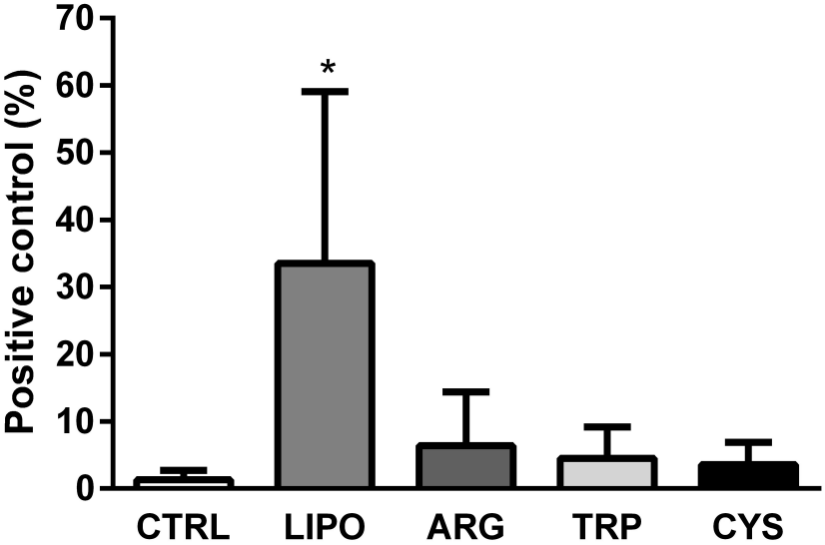
*****eGFP*** gene delivery on lung tissue**. eGFP RNA levels measured in the lungs of animals that received liposomes conjugated with a DNA plasmid containing the *eGFP* gene (LIPO, ARG, TRP, and CYS groups), or the free DNA plasmid in vehicle (CTRL). Values are given as percentage of expression in relation to positive eGFP transgenic animals samples (mean ± *SD*). ^*^Significantly different from the CTRL, ARG, TRP, and CYS groups (*P* < 0.05).

In order to verify the efficacy of the *eGFP* gene delivery by these lipopolymers when conjugated or not with amino acids, we performed a quantitative RT-PCR assay on lung samples of treated animals. We observed that there was accumulation of mRNA for eGFP in all animals that received the lipopolymers containing the *eGFP* plasmid. Conversely, the animals that received only the free DNA plasmid did not show eGFP mRNA levels. Furthermore, lipopolymers that did not contain amino acids had a better efficacy in gene delivery than the arginine-, cysteine-, and tryptophan-conjugated liposomes (Figure 7).

## Discussion

In this study, we developed lipopolymeric particles associated with three different amino acids, and showed that amino acid integration has different effects on polymerization, according to the structure and net charge of the amino acid, but does not halt the formation of lipopolymers. The amino acids were selected based on their positive charge and better DNA association (l-arginine), their hydrophobic characteristics that have an influence on liposome stability (l-tryptophan; Fernandez-Ruocco et al., 2013), and their ability to interact and reduce the resistance of the mucus barriers (l-cysteine; Poelma et al., 1990).

According to the data obtained in the efficiency polymerization assay and HF determination (Figures 2, 3), l-arginine induces the highest number of polymeric units as a result of a combination of electrostatic and hydrophobic interactions, because l-arginine induced the lowest HF compared with the other amino acids. l-Tryptophan has only a hydrophobic interaction and it can induce a decrease in HF, but this decrease is lower than that observed for l-arginine. l-Cysteine cannot establish efficient hydrophobic or electrostatic interactions, so it has the lowest encapsulation efficiency and it is released rapidly. Furthermore, we observed that l-tryptophan could establish an ideal membrane surface rigidity providing sustained release over time.

The difference in the concentration of the polymer units generated by l-arginine could be explained by the differences in net charge. In a study conducted by Wang et al. (2008), it has been reported that the phase of a lipid bilayer composed of phospholipids with phosphocholine heads can be modified locally by a nanoparticle loaded with a specific charge. Phosphocholine heads have an electric dipole choline phosphate (P^−^ N^+^). In the presence of charged particles, this dipole changes its angle generating local phase changes. When the nanoparticle has a net positive charge, such as in l-arginine, the phosphocholine head tilts its usual angle of 30° to an angle of ~65° characteristic of the gel phase, inducing an increased level of packing. l-Tryptophan and l-cysteine have no net charges, therefore, do not produce this effect on phosphocholine heads, giving a smaller number of polymer units as a result.

Amino acids have been used extensively in biotechnology in the production of pharmaceuticals due to their natural ability to stabilize proteins and their adjuvant properties (Arakawa et al., 2007); they are of great interest for their physiologic effects. l-Cysteine is known to be important for response to oxidative stress, because it is required for the synthesis of glutathione, and exerts beneficial effects in oxidative stress in lung tissue (Métayer et al., 2008; Mendoza et al., 2013). l-Arginine, a nitric oxide precursor, has positive effects on respiratory illness, such as post-cardiopulmonary bypass lung injury (Chao et al., 2011) and asthma (Mabalirajan et al., 2010; Arıkan-Ayyıldız et al., 2014). Besides its important role as a precursor of neurotransmitters, l-tryptophan is metabolized by the enzyme indoleamine 2,3-dioxygenase, produced by dendritic cells and macrophages, as an immunosuppressive mechanism, which forms catabolites that reduce T-cell proliferation and survival (Munn and Shafizadeh, 1999; Fallarino et al., 2002).

We noticed that no toxicity was observed *in vitro* using the Caco-2 cell line and the MTT method [9,37] (data not shown), and therefore we moved forward to test the toxicity of these particles *in vivo*.

Using a dose of 2 mg/ml, no morphologic or mechanical changes were observed in the lungs of the animals and the number of inflammatory cells in the lung tissue was not altered, suggesting that these particles are safe for local delivery into the lungs, independent of conjugation with or without amino acids.

Gene delivery is a major challenge in the treatment of respiratory diseases, especially with regard to the development of vectors. Most lung gene therapy trials have used viral gene vectors, but these vectors have shortcomings, such as inflammatory and immune responses of the host against the viral particles acutely and on sensitization when multiple doses are required, and limited plasmid size (Gautam et al., 2012). Among non-viral vectors, cationic liposomes have been studied extensively and are promising vectors for pulmonary gene delivery (Simões et al., 2005; Cipolla et al., 2014). The size, composition, and structure of the nanoparticles are essential for their efficacy. Polymerized liposomes, composed of the photopolymerizable lipid DC_8, 9_PC) are more stable in different media compared with the non-polymerized formulations, and do not develop cell toxicity after polymerization (Alonso-Romanowski and Chiaramoni, 2003; Yavlovich et al., 2009). However, intravenous or local delivery of cationic liposomes carrying DNA has led to inflammatory responses in several instances (Freimark et al., 1998; Alton et al., 1999). Therefore, further development of less immunogenic, non-toxic liposome carriers is of great interest.

We have found that polymerized liposomes composed of DMPC/DP_8, 9_PC, without conjugation with amino acids, were efficient in causing gene expression in lung tissue, leading to >30 times higher expression of the *eGFP* gene compared with the delivery of naked DNA. Furthermore, this ability is impaired after conjugation with the amino acids, suggesting that the DNA interacts with the polar heads of the lipids, and this interaction is halted by the presence of amino acids in these sites (Figure 7).

From these results, we can state that for the delivery and expression of DNA to lung tissue, the best formulation is the one developed with polymeric liposomes with no amino acids added, because for this route of administration they added no benefits.

The sustained expression of the delivered gene remains to be evaluated; here, we analyzed a single time point (24 h) after transfection. In addition, further toxicity experiments are needed, given that lipid/DNA complexes might present toxicity even when the unconjugated liposomes do not cause significant harm (Tousignant et al., 2000).

The characterization of the liposomes, taken together with the efficacy in delivery of the eGFP gene to the lung tissue of healthy mice, prompt us to suggest that these liposomes are an interesting, stable alternative to be tested as gene delivery or drug delivery carriers and adjuvants directly into the lungs.

## Author contributions

EB—acquired, analyses and interpretation of data, drafting, and revising the work for important intellectual content; ML—acquired, analyses and interpretation of data, drafting and revising the work, experimental design and organization; NC—analyses and interpretation of data, drafting the manuscript; DF—data acquistion, revised the work for important intelectual content; MF—data acquistion, revised the work for important intelectual content; MP—data acquistion, revised the work for important intelectual content; TM—data acquistion, revised the work for important intelectual content; RP—data acquistion, revised the work for important intelectual content; HC—experimental design and organization, interpretation of data, revised the work for important intellectual content; PR—experimental design and organization, interpretation of data, revised the work for important intellectual content; SA—experimental design and organization, revised the work for important intellectual content; MM—experimental design and organization, interpretation of data, revised the work for important intellectual content. All authors approved final version to be published, agreement to be accountable for all aspects of the work in ensuring that questions related to the accuracy or integrity of any part of the work are appropriately investigated and resolved.

### Conflict of interest statement

The authors declare that the research was conducted in the absence of any commercial or financial relationships that could be construed as a potential conflict of interest.
